# Resistance to Thrips in Peanut and Implications for Management of Thrips and Thrips-Transmitted Orthotospoviruses in Peanut

**DOI:** 10.3389/fpls.2018.01604

**Published:** 2018-11-06

**Authors:** Rajagopalbabu Srinivasan, Mark R. Abney, Pin-Chu Lai, Albert K. Culbreath, Shyam Tallury, Soraya C. M. Leal-Bertioli

**Affiliations:** ^1^Department of Entomology, University of Georgia, Griffin, GA, United States; ^2^Department of Entomology, University of Georgia, Tifton, GA, United States; ^3^Department of Plant Pathology, University of Georgia, Tifton, GA, United States; ^4^United States Department of Agriculture – Agricultural Research Service, Griffin, GA, United States; ^5^Department of Plant Pathology, University of Georgia, Athens, GA, United States

**Keywords:** *Frankliniella fusca*, peanut, resistance, wild species, vector, *Orthotospovirus*

## Abstract

Thrips are major pests of peanut (*Arachis hypogaea* L.) worldwide, and they serve as vectors of devastating orthotospoviruses such as *Tomato spotted wilt virus* (TSWV) and *Groundnut bud necrosis virus* (GBNV). A tremendous effort has been devoted to developing peanut cultivars with resistance to orthotospoviruses. Consequently, cultivars with moderate field resistance to viruses exist, but not much is known about host resistance to thrips. Integrating host plant resistance to thrips in peanut could suppress thrips feeding damage and reduce virus transmission, will decrease insecticide usage, and enhance sustainability in the production system. This review focuses on details of thrips resistance in peanut and identifies future directions for incorporating thrips resistance in peanut cultivars. Research on thrips–host interactions in peanut is predominantly limited to field evaluations of feeding damage, though, laboratory studies have revealed that peanut cultivars could differentially affect thrips feeding and thrips biology. Many runner type cultivars, field resistant to TSWV, representing diverse pedigrees evaluated against thrips in the greenhouse revealed that thrips preferred some cultivars over others, suggesting that antixenosis “non-preference” could contribute to thrips resistance in peanut. In other crops, morphological traits such as leaf architecture and waxiness and spectral reflectance have been associated with thrips non-preference. It is not clear if foliar morphological traits in peanut are associated with reduced preference or non-preference of thrips and need to be evaluated. Besides thrips non-preference, thrips larval survival to adulthood and median developmental time were negatively affected in some peanut cultivars and in a diploid peanut species *Arachis diogoi* (Hoehne) and its hybrids with a Virginia type cultivar, indicating that antibiosis (negative effects on biology) could also be a factor influencing thrips resistance in peanut. Available field resistance to orthotospoviruses in peanut is not complete, and cultivars can suffer substantial yield loss under high thrips and virus pressure. Integrating thrips resistance with available virus resistance would be ideal to limit losses. A discussion of modern technologies such as transgenic resistance, marker assisted selection and RNA interference, and future directions that could be undertaken to integrate resistance to thrips and to orthotospoviruses in peanut cultivars is included in this article.

## Introduction

### Thrips Feeding Damage and Virus Transmission in Peanut

**FIGURE 1:**
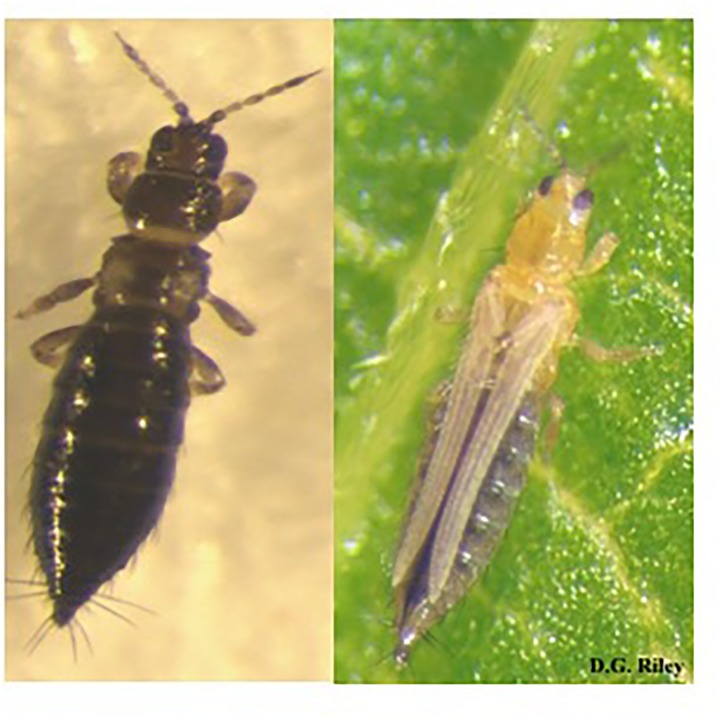
Two common thrips species in peanuts in Southeastern United States, tobacco thrips, *Frankliniella fusca* (Hinds), on the left, and the western flower thrips, *Frankliniella occidentalis* (Pergande), on the right.

Peanut (*Arachis hypogaea* L.) is a major food and oil seed crop that provides high quality human nutrition and is severely affected by thrips and viruses transmitted by them in many parts of the world including the Southern United States, South/Southeastern Asia, and South America (Pappu et al., 2009; Riley et al., 2011; Mandal et al., 2012). Thrips are small (<2 mm in length) and slender insects with fringed wings belonging to the order Thysanoptera. They are hemimetabolous insects with egg, larvae, prepupal (quasi pupal stage), and adult stages. The adults and larvae are the two mobile stages, with adults alone possessing wings (Lewis, 1973, 1997). The two common wing morphs include the brachypterous (short-winged) and macropterous (long-winged) forms. Depending upon seasonal environmental parameters and host availability, thrips alternate wing forms to aid their dispersal. In the United States, two thrips species, Western flower thrips, *Frankliniella occidentalis* (Pergande), tobacco thrips, *Frankliniella fusca* (Hinds), occur in most peanut producing areas (Figure 1; Sakimura, 1963; Todd et al., 1995; Riley et al., 2011). In Southeastern United States, where more than half of United States peanuts are grown, *F. fusca* is commonly found on peanut foliage and flowers, and is responsible for almost all the early season feeding injury in peanut (Todd et al., 1995). Western flower thrips is predominantly a flower feeder, and is often found later in the growing season. In other peanut growing areas, such as South and Southeast Asia, thrips species *viz*., common blossom thrips, *Frankliniella schultzei* (Trybom), chili thrips, *Scirtothrips dorsalis* (Hood), melon thrips, *Thrips palmi* (Karny), bean flower thrips, *Megalurothrips usitatus* (Bagnall), and groundnut thrips, *Caliothrips indicus* (Bagnall) are known to infest peanut (Amin et al., 1985; Ekvised et al., 2006a). In South America, besides *Frankliniella* sp. others such as *Enneothrips flavens* (Moulton) are commonly found on peanuts (de Souza et al., 2010; Michelotto et al., 2017).

**FIGURE 2:**
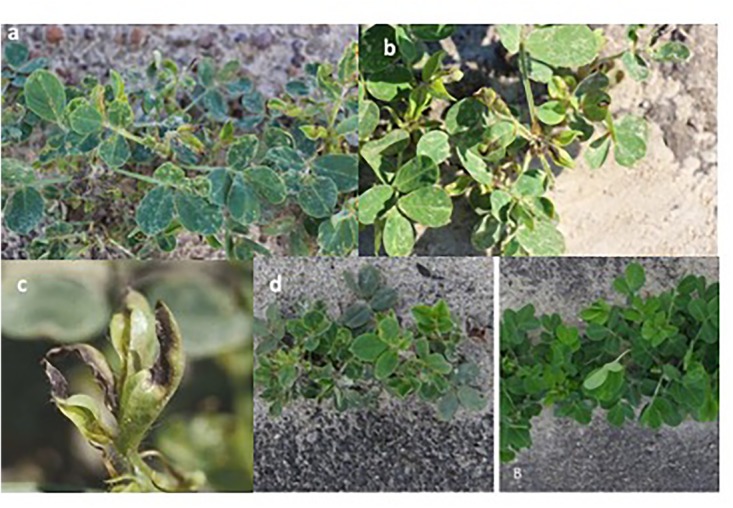
Thrips induced feeding symptoms on peanut. Silvering appearance due to thrips feeding on the epidermal cells’ contents **(A)**, leaf-tip yellowing and necrosis and curling of newly developing leaflets (terminals) at the shoot tip **(B)**, a close-up view of tip burning in terminal leaflets **(C)**, and stunting of thrips-infested non-treated peanut plant on the left and treated peanut plant on the right **(D)**.

Thrips feeding in peanut is a concern from the time of seedling emergence to a few weeks following emergence. Under severe thrips pressure, thrips feeding injury early in the season can result in yield loss and/or delayed maturity (Todd et al., 1995; de Moraes et al., 2005; Funderburk et al., 2007). Thrips possess asymmetrical mouthparts, due to an atrophied mandible, and generally feed by sucking plant cell contents. In the process, thrips feeding is often characterized by “silvering” of leaves. The silvering appearance is caused by empty epidermal cells following thrips feeding on the cells’ contents (Figure 2A). Larvae and adults can feed on the peanut foliage. When thrips populations are high early in the growing season, a situation characterized by extensive larval colonization, it is common to find leaf-tip yellowing and necrosis and curling of newly developing leaflets (terminals) at the shoot tip (Figures 2B,C). Heavily infested peanut seedlings are often stunted and in severe cases can die (Figure 2D). Thrips feeding also results in transmission of viruses. Orthotospoviruses are a major peanut-infecting virus group that is of concern worldwide (Jones, 2005; Pappu et al., 2009; Riley et al., 2011). The main orthotospoviruses (Family *Tospoviridae*; Order *Bunyavirales*) include the *Tomato spotted wilt virus* (TSWV) in Asia and in North America, *Groundnut ring spot virus* (GRSV) and *Groundnut bud necrosis virus* (GBNV) in Asia, and GRSV in South America (Pappu et al., 2009). Increased thrips populations are often correlated with increased virus incidence (Garcia et al., 2000; Culbreath et al., 2003; Sharma et al., 2003).

### Thrips Management Options in Peanut and Limitations

Thrips employ haplodiploid sex determination, wherein the fertilized eggs produce diploid females, and the non-fertilized eggs result in haploid males (Moritz, 1997). This mode of reproduction and rapid life cycle (dependent on temperature) allows them to build up in large numbers. Their populations are usually characterized by one or two peaks in a typical peanut growing season. Their reproductive capacity, short lifecycles, broad host range, and thigmotactic behavior (seeking refuge in tight spaces such as unfolded peanut terminals), make thrips difficult to manage in peanut. Cultural practices can significantly affect thrips populations in peanut. Manipulating planting date to avoid coincidence of peak thrips dispersal and the susceptible seedling stage results in lower thrips densities and reduces the risk of feeding injury and virus transmission (Brown et al., 1996; McKeown et al., 2001; Culbreath et al., 2010). Likewise, seeding into heavy plant residue in conservation tillage systems reduces thrips abundance on peanut compared with conventional tillage systems with bare soil (Brown et al., 1996; Monfort et al., 2007). Increased plant density and twin row planting have also been shown to reduce thrips infestation and virus incidence, though the mechanism(s) responsible are not well understood (Culbreath et al., 2008; Tubbs et al., 2011). Unfortunately, all the management tactics discussed here have potential negative consequences. Planting dates that minimize risk of thrips infestation are not always optimal for maximizing yield. While conservation tillage offers several recognized agronomic and environmental benefits in addition to thrips management, it may have negative effects on weed management programs compared with conventional tillage systems (Johnson et al., 2001). The additional seed and specialized planting equipment needed to increase plant densities and achieve twin row patterns ultimately increase production costs for farmers.

Chemical management options for thrips in peanut, like many other row crops, are limited to a few insecticide active ingredients (Reddy et al., 1995; Todd et al., 1995, 1996; Herbert et al., 2007; Culbreath et al., 2008; Marasigan et al., 2016). The most commonly used insecticide classes include organophosphates, carbamates, phenylpyrazole, pyrethroids, and neonicotinoids (Todd et al., 1996; Culbreath et al., 2003, 2016; Mandal et al., 2012; Marasigan et al., 2016; Srinivasan et al., 2017). Newer classes of insecticides such as spinosyns and diamides, though effective, are too expensive to justify their use in peanut (Marasigan et al., 2016, 2018). These products are generally reserved for high value crops such as fruits and vegetables. In addition, there is increased concern over environmental and non-target issues associated with older broad-spectrum insecticides such as organophosphates, and carbamates. Even neonicotinoids, long considered to be reduced risk options, are being scrutinized due to their presumed role in pollinator decline (Mullin et al., 2010; Nicodemo et al., 2014). In lieu of the perceived effects, the United States Environmental Agency in 2015 issued a temporary moratorium on new neonicotinoid registrations^1^. This moratorium has not affected any existing registrations. Neonicotinoids applied as liquid in-furrow at peanut planting offer ease of application, are relatively less expensive, and in general provide good efficacy. For these reasons, the neonicotinoid imidacloprid is now used increasingly in Georgia in Southern United States (Marasigan et al., 2016, 2018; Srinivasan et al., 2017). Thrips, in general, have effective pesticide detoxification abilities (Espinosa et al., 2005; Bielza et al., 2007, 2008; Bielza, 2008), and they have developed resistance to several insecticide classes. Increased neonicotinoid usage in cotton has already led to widespread resistance development in the Southeastern United States (Huseth et al., 2016). Preliminary research conducted in Georgia in the United States indicated no evidence of resistance to neonicotinoids in thrips populations collected from peanut (Lai, 2015). The usefulness of these insecticides in the long run remains questionable. Of course, the main concern in peanut production in many parts of the world is thrips-transmitted orthotospoviruses. No insecticide, except phorate, has been found to be effective in suppressing virus transmission significantly in the United States, and the effect of phorate is not consistent (Culbreath et al., 2003, 2008, 2016; Marasigan et al., 2018).

Because of the difficulty associated with managing thrips and the significant economic loss that accompanies virus infection, a tremendous amount of effort has been invested into breeding cultivars with resistance against orthotospoviruses. Much of the information on breeding for virus resistance comes with research conducted with TSWV in peanut in the United States and GBNV in Asia. The cultivars grown at the advent of TSWV in Southern United States, in the 1980s and in early 1990s, such as Florunner and Southern runner, were extremely susceptible to TSWV-induced spotted wilt disease (Culbreath et al., 1992, 1996). Screening and breeding efforts led to incremental increases in resistance, most of which was derived from a single genotype (PI 203363) introduced from Brazil in 1953 (Culbreath et al., 2003). The introduction of this unique genotype had a rapid and profound effect on the United States peanut breeding, as the main runner peanut cultivars have a significant proportion of PI203363 alleles (Clevenger et al., 2018). Current “third generation TSWV-resistant” peanut cultivars are highly field resistant to TSWV, and losses due to the disease have been minimized. Breeding efforts also have led to identification of moderate resistance to GBNV in Asia (Amin et al., 1985; Dwivedi et al., 1995; Reddy et al., 2000; Kesmala et al., 2004; Mandal et al., 2012). In all these instances, resistance to the virus is not complete, and often other management options are integrated. For instance, insecticides are still being employed to reduce thrips feeding injury in early season peanut (Mandal et al., 2012; Marasigan et al., 2016). Identifying and incorporating effective thrips resistance in high yielding peanut cultivars will provide significant economic benefit to producers and result in reduced environmental impact associated with pesticide use.

### Factors Contributing to Resistance Against Thrips in Peanut in Relation to Other Crops

Thrips resistance in peanut was more actively pursued in the 1980s and early 1990s in Asia and in the United States until thrips-transmitted viruses became a more pressing issue (Amin and Mohammed, 1980; Campbell and Wynne, 1980; Amin et al., 1985; Mulder and Seuhs, 2002). Most of those early examinations were based on field screening (Young et al., 1972; Stalker and Campbell, 1983; Lynch, 1990). Consequently, thrips resistance contributing factors in peanut are not well understood. Information about thrips resistance stems mostly from work on other crops, where resistance seems to be imparted by morphological as well as biochemical traits. Each trait category is discussed in detail.

#### Morphological Traits

Early on increased leaf pubescence in crops such as cassava was associated with thrips resistance (Schoonhoven, 1974). Similarly, increased foliar pubescence in diploid cotton such as *Gossypium arboreum* L., *Gossypium thurberi* (Todd), *Gossypium trilobum* (DC.) Skovst resulted in reduced western flower thrips infestation compared with other commonly grown *Gossypium hirsutum* L. cv. Sicot 71 (Miyazaki et al., 2017). Another study found that thrips infestation was less in glandless cotton than in glandular cotton (Zhang et al., 2014). Increased leaf waxiness was associated with resistance against thrips in cabbage (Voorrips et al., 2008a,b). On the contrary, in onion cultivars, glossy (less wax) yellow green foliage provided more protection against thrips than cultivars with non-glossy or waxy blue green foliage (Coudriet et al., 1979; Molenaar, 1984; Diaz-Montano et al., 2010; Damon et al., 2014). Traits such as leaf angle and leaf toughness were also more influential than waxiness on host plant susceptibility to onion thrips (Njau et al., 2017). Onion foliage that was more open and round, allowed more thrips exposure to natural enemies, and thus had fewer thrips compared with onion foliage that was tight and had short angle of deviation. In addition to leaves, floral, and fruiting structures are also vulnerable to thrips. It has been shown in at least two cases that the size of floral structures can be associated with thrips resistance. Smaller flowers in both cowpea and chrysanthemum resulted in reduced incidences of *Megalurothrips sjostedti* (Trybom) and *F. occidentalis*, respectively (de Jager et al., 1995; Abudulai et al., 2006; Omo-Ikerodah et al., 2009).

Field screening of peanut genotypes revealed differences in thrips feeding injury consistently, and it was speculated that foliage color could be influencing thrips host selection patterns (Amin et al., 1985). A relatively recent study found differences in normalized vegetation index among peanut cultivars, which in turn might affect light reflectance off peanut foliage (Navia-Gine, 2012). It is not clear if these differences in light reflectance affect thrips host plant utilization. The hypothesis that reflectance contributes to host plant resistance should be evaluated, and the role of light reflectance in thrips host selection and host utilization in peanut needs to be examined in greater depth. Such differences in foliar reflectance in light could be due to differences in profiles of cuticular waxes. Peanut cultivars’ and wild species foliage hues differ substantially from light green to bluish green. Increased cuticular wax in various wild peanut species such as *Arachis batizocoi* Krapov. & W. C. Gregory, *Arachis glandulifera* Stalker, *Arachis ipaensis* Krapov. & W. C. Gregory, *Arachis Chacoense* Krapov., and *Arachis paraguariensis* Chodat & Hassl., is believed to be responsible for suppressing thrips feeding compared with commonly grown peanut cultivars (Yang et al., 1993; de Souza et al., 2010). Other morphological traits discussed previously such as leaf hairiness, and leaf toughness are also believed to be involved in conferring resistance to insects including thrips in peanut (Campbell and Wynne, 1980; Yang et al., 1993), but their role in suppressing thrips needs to be experimentally demonstrated.

#### Biochemical Traits

Alkaloids and other secondary metabolites seem to be contributing to thrips resistance either in the presence or absence of morphological traits. In a study with wild tomato species *viz*., *Solanum pennellii* Correll and *Solanum hirsutum* Dunal, acyl sugars were implicated as being involved in conferring resistance to thrips (Mirnezhad et al., 2010; Romero González, 2011). An alkaloid, pyrrolizidine, was associated with thrips resistance in the case of *Jacobaea aquatica* (Hill) G. Gaertn., (Cheng et al., 2011). Similarly, glycoalkaloids provided resistance against thrips in potato, *Solanum tuberosum* L. (Galvez et al., 2005). Evidence for involvement of biochemical compounds besides alkaloids in thrips resistance is found in numerous crops. Isobutylamides of unsaturated fatty acids and chlorogenic acid from chrysanthemum, *Chrysanthemum indicum* L., conferred resistance against western flower thrips (Tsao et al., 2003; Leiss et al., 2009, 2011). A flavonoid luteolin and phenylpropanoid sinapic acid derived from carrot, *Daucus carota* (Hoffm.) Schübl., negatively impacted Western flower thrips development (Leiss et al., 2013). In some instances, biochemicals are thought to be involved in imparting resistance against thrips; however, they have not been identified. For instance, steam distillates from certain resistant cultivars of rice (*Oryza indica* L.) were toxic to *Stenchaetothrips biformis* (Bagnall), but it is not clear which active compound(s) in that distillate was responsible for thrips mortality (Velusamy and Saxena, 1991). Similarly, extensive studies in pepper, *Capsicum* sp., led to identification of resistance believed to be caused by biochemical traits, but the causal agents have not been identified (Maris et al., 2003a,b; Maharijaya, 2013). Also, some odors from rose, *Rosa* sp., that repelled western flower thrips, and are yet to be characterized (Gaum et al., 1994). de Souza et al. (2010) found that certain n-alkanes from wild diploid peanut species could be responsible for *E. flavens* resistance in Brazil. A polyphenolic compound, 2,3-Di-(E)-caffeoyl-(2B,3R)-(+)-tartaric acid, found in peanut terminals was associated with *F. fusca* resistance in the Southeastern United States (Snook et al., 1994). However, this study merely reported a correlation, and did not present a conclusive evidence of the compound’s involvement in resistance against thrips. Another study showed a strong negative correlation between phenols and tannins in peanut germplasm with thrips feeding damage (Kandakoor et al., 2014). The correlative roles of these compounds in thrips resistance should further be functionally characterized by isolating these compounds and conducting bioassays with thrips.

### Mechanism(s) of Resistance Against Thrips in Peanut

**FIGURE 3:**
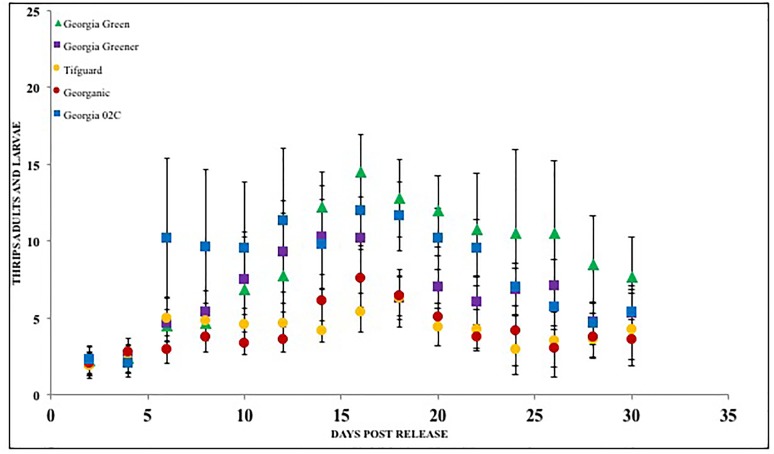
Thrips larvae and adults counted on peanut foliage of various cultivars that are resistant and susceptible to *Tomato spotted wilt virus* under a choice situation. Georgia Green is considered as a TSWV-susceptible cultivar. Thrips counts were taken at two-day intervals since release, and counted for 3 weeks post initial thrips release.

Peanut cultivars have been screened for *F. fusca* and *F. Schultzei* feeding injury in numerous studies in the Southern United States, Asia, and in South America (Young et al., 1972; Kinzer et al., 1973; Amin et al., 1985; Sharma et al., 2003). In the United States, several studies identified runner type and Virginia type peanut germplasm plant introductions with resistance to thrips in Georgia and North Carolina (Lynch, 1990). Results from these studies showed differences in thrips injury rating. However, the peanut plants generally recovered from thrips injury and yield losses only occurred under certain conditions (Funderburk et al., 2007). The losses that were observed were more often associated with Virginia type peanut cultivars than runner type (Amin and Mohammed, 1980; Campbell and Wynne, 1980; Mulder, 1999; Mulder and Seuhs, 2002; Herbert et al., 2005; Whalen et al., 2014). Choice experiments conducted with peanut cultivars and tobacco thrips suggest that cultivars may differently affect thrips density as well as severity of thrips feeding injury (Figure 3). Thrips feeding was reduced in some cultivars such as “Tifguard” and “Georganic” when compared with others such as Georgia Green and Georgia 06G (Sundaraj et al., 2014). Thrips feeding was measured by using a thrips feeding damage index, which is a measurement of silvering area on the plant as a proportion of the undamaged area (Maris et al., 2003a). The evaluated peanut cultivars were actually released with increased resistance to TSWV, but none were specifically bred for thrips resistance. These results suggest that there could be non-preference or antixenosis effects present in peanut cultivars that impact the behavior of thrips. The term non-preference was defined by Painter (1951); Kogan and Ortman (1978) later described it as antixenosis, these terms describe the inability of the insect to effectively use a host plant and instead select an alternate host plant (Smith, 2005). It is not clear how these preference patterns would influence thrips in peanut agroecosystems with relatively low genotype diversity such as those in the Southern United States. For instance, in Georgia, more than 80% of the peanut acreage (>600 K acres, NASS, 2017) is often planted with a single cultivar. More research needs to be conducted to evaluate the significance of antixenosis against thrips in commercial peanut production.

**FIGURE 4:**
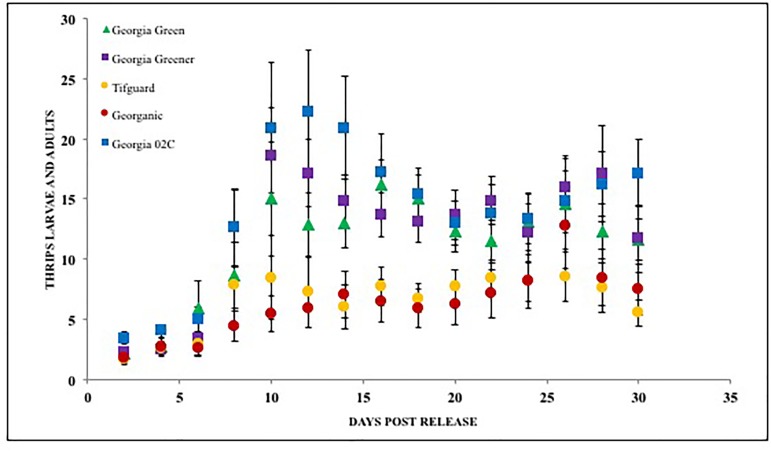
Thrips larvae and adults counted on peanut foliage of various cultivars that are resistant and susceptible to *Tomato spotted wilt virus* under a no-choice situation. Georgia Green is considered as a TSWV-susceptible cultivar. Thrips counts were taken at two-day intervals since release, and counted for 3 weeks post initial thrips release.

**FIGURE 5:**
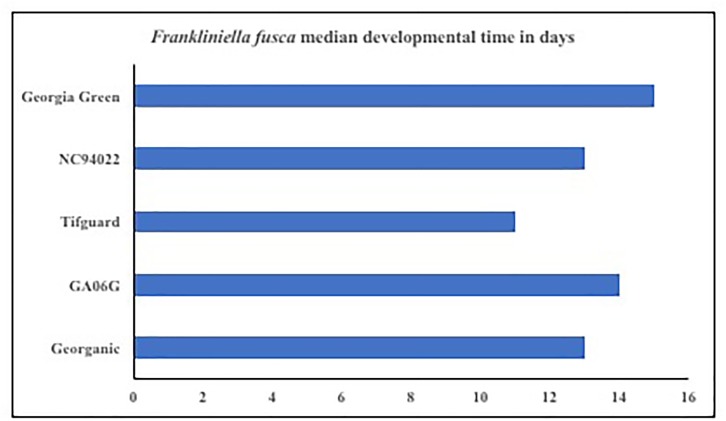
Thrips developmental time on *Tomato spotted wilt virus* resistant and susceptible cultivars. Developmental time refers to the time take from egg to adulthood, and counts were taken once every 2 days. Thrips developmental time was monitored using Plexiglass arenas called Munger cages.

Microcosmic “Munger”(45 cm^3^ thrips-proof cages) studies indicate that peanut cultivar differences could differentially influence tobacco thrips, *F. fusca* fitness. No-choice tests to monitor thrips development and thrips survival were conducted with commonly grown cultivars in Georgia. Results revealed that cultivar differences significantly influenced tobacco thrips survival (Figure 4; Sundaraj et al., 2014). Thrips fitness was consistently reduced in some of the “second-generation TSWV resistant” peanut cultivars compared to ones released earlier such as “Georgia Green.” Antibiosis is a resistance mechanism by which a host plant adversely affects the biology of the insect, often resulting in increased mortality or reduced longevity and fecundity (Teetes, 1996). The Munger cage studies revealed that thrips fecundity (adults recovered per adult released) and longevity was reduced in some cultivars such as Georganic and Tifguard. The innate factors in these cultivars that contribute to antibiosis against thrips are yet to be identified. Unique parentage of these cultivars could be influencing resistance to thrips. For instance, “Tifguard,” which possesses resistance to nematodes seems to be the most resistant against thrips (Holbrook et al., 2008; Shrestha et al., 2013). Nematode resistance in Tifguard is derived from a germplasm accession line “COAN”; it is not clear if the nematode resistance and thrips resistance are interlinked (Holbrook et al., 2008). Fitness experiments also revealed that the median thrips developmental time from egg to adult in Tifguard was lower than in any other cultivar evaluated (Figure 5; Shrestha et al., 2013). The reduced developmental time (by 2 days) of *F. fusca* on a resistant cultivar could be a strategy used by thrips to overcome unfavorable characteristics in that resistant genotype. This strategy is not unique to *F. fusca* and peanut; variations in thrips developmental time have been associated with *F. occidentalis* on thrips-resistant pepper (Maris et al., 2003b; Maharijaya et al., 2012). This phenomenon has also been observed in other insects (Leather et al., 1998).

Breeding efforts for thrips resistance in peanut has dwindled in the United States and continues on a minor scale in Asia Culbreath et al., 2003; Pappu et al., 2009; Culbreath and Srinivasan, 2011; Mandal et al., 2012; Srinivasan et al., 2017). A study on heritability of thrips resistance in Thailand found that there was a weak correlation between thrips resistance parameters and agronomic traits and predicted that these characters are independently inherited (Ekvised et al., 2006b). With increased adoption of TSWV and other virus-resistant cultivars in most peanut production areas, there is heightened concern about evolution of resistance breaking virus strains (Sundaraj et al., 2014). Prior evidence of TSWV resistance breakdown in solanaceous crop hosts should serve as a warning (Qiu and Moyer, 1999), and all protections should be taken to prevent virus-resistance breakdown in peanut cultivars. Evidence presented above suggests that peanut cultivar-thrips interactions could influence virus transmission by thrips (Sundaraj et al., 2014). Results from Sundaraj et al. (2014) revealed that some of the TSWV-resistant cultivars such as Tifguard and Georganic accumulated less viral copies (up to 1/3rd less) and were infected at a lower percentage (up to 20%) than other TSWV resistant cultivars such as Georgia Green and Georgia Greener. These results suggest that cultivars that negatively affect thrips preference and/or fitness further suppress virus incidence and accumulation in cultivars that are already moderately resistant to TSWV, thereby providing additive effects. Therefore, it is logical to assume that stacking thrips resistance with virus resistance would reduce the amount of selection pressure imparted against the virus itself, delay the development of resistance breaking strains, and prolong the usefulness of these resistant cultivars that are heavily relied upon. In the United States in Georgia and North Carolina, more than 80% of the peanut acreage is planted with TSWV-resistant cultivars. Losing TSWV resistance in these cultivars would be devastating to peanut production. One way to prevent such a resistance breakdown would be to identify and incorporate thrips resistance in conjunction with TSWV resistance.

## Role of Peanut Wild Relatives for Resistance

Wild species of peanut have been sources of resistance for many pests and diseases in peanut. Resistance to thrips is no exception. The most remarkable being the production of varieties with resistance to root-knot nematode, leaf spot, and to rust, all derived from wild species (Simpson, 1991; Simpson et al., 2003; Khedikar et al., 2010; Chu et al., 2011). Several wild species have been associated with thrips resistance in peanut. Stalker and Campbell (1983) evaluated more than 30 wild species of *Arachis* over 3 years through field screening and identified several species that possess resistance to thrips. The number of thrips damaged leaves per plot on *A. batizocoi*, *Arachis correntina* (Benth.), *Arachis villosa* Benth., *Arachis spegazzini* Greg., *A. chacoense, Arachis cardenasii* Krapov., *A. stenosperma* Krapov. & W. C. Greg., *Arachis duranensis* Krapov, *Arachis rigonii* Krapov., *Arachis paraguariensis* Chodat & Hassl., *Arachis pusilla* Benth., and *Arachis repens* Handro, were two to ∼100 times less than on peanut cultivars. Similar evaluations were conducted in other places in the Southern United States as well as in Asia, and several genotypes were found to possess thrips resistance (Amin and Mohammed, 1980; Lynch, 1990). Diploid wild species such as *Arachis vallsii* Krapov. & W. C. Greg., *Arachis kempff-mercadoi* Krapov., W. C. Greg. & C. E. Simpson, *Arachis williamsii* Krapov. & W. C. Greg., *A. duranensis*, and amphidiploids such as *A. batizocoi × A. kempff-mercadoi, A. gregoryi × A. stenosperma, A. magna × A. cardenasii* also exhibited substantial levels of resistance to *E. flavens* in South America (Michelotto et al., 2017). Most of these evaluations were based on field screening, and the mechanism by which resistance is conferred in some of these wild species is not known. Few studies have examined the resistance contributing factors in these wild species. Epicuticular waxes containing n-alkanes from *A. batizocoi, A. chacoense, A. paraguariensis, A. glandulifera*, and *A. ipaensis* were speculated to confer resistance to thrips and other sucking insects, probably through non-preference/antixenosis (Yang et al., 1993). The same group of compounds, were also identified in *Arachis monticola* Krapov. & Rigoni and *A. stenosperma*, through gas chromatography (de Souza et al., 2010). In Brazil, another study showed that various wild species exhibited field resistance to *E. flavens* (Michelotto et al., 2017). For a more comprehensive list of wild species with resistance to thrips and TSWV, please refer to review by Stalker (2017).

**FIGURE 6:**
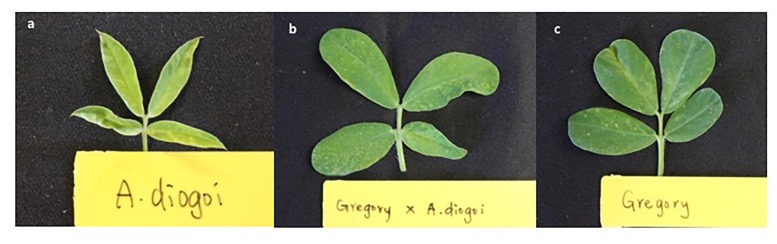
Leaf phenotype of a diploid wild species, *Arachis diogoi* **(A)**, cultivated tetraploid *Arachis hypogaea* cultivar Gregory **(C)**, and a cross between the two **(B)**.

While there is evidence that wild species exhibit resistance against thrips, their ploidy level, makes it difficult to incorporate their resistance into cultivated tetraploid peanuts. Most wild species are diploids. Resistance introgression from wild species is achieved by crossing and subsequent backcrossing. This process is time consuming, and a major concern has been the reduction of level of resistance while backcrossing. *Arachis diogoi* is a diploid wild species that is known to possess resistance to numerous pests and pathogens including TSWV (Lyerly et al., 2002). Attempts were made to introgress TSWV resistance from *A. diogoi* to the Virginia type cultivar Gregory, using the hexaploid route (Stalker HT et al., 1979; Figure 6). Several progenies from those crosses accumulated fewer copies of the virus when compared with Gregory (Lai, 2015). Subsequently evaluations for thrips resistance were done using microcosmic Munger cages (Lai, 2015). Results reiterated that *A. diogoi* was highly resistant to thrips when compared with the cultivar Gregory; however, the genotypes from the intraspecific crosses were not anymore resistant than the recurrent parent Gregory (Lai, 2015). Thrips fitness parameters such as developmental time and fecundity were evaluated, and thrips developmental time was (20%) longer and its fecundity was lower on *A. diogoi* than on tetraploid Gregory. These results revealed direct effects on thrips biology suggesting that there could be antibiosis-based resistance against thrips.

A different route of introgression, called the tetraploid route involves crossing two wild species of complementary genomes and doubling the resultant sterile diploid hybrid with colchicine (Simpson, 1991; Leal-Bertioli et al., 2012, 2015). The resultant synthetic allotetraploid possesses all genes from both wild species and is compatible with peanut, being, therefore, useful for breeding. In Brazil, synthetics developed using this strategy were evaluated for field resistance to thrips, and three of them [(*A. batizocoi* × *A. kempff-mercadoi*)^4x^, (*A. gregoryi* × *A. stenosperma*)^4x^, and (*A. magna* × *A. cardenasii*)^4x^] were found to have superior resistance than the cultivars tested, and are, therefore potentially useful for breeding (Michelotto et al., 2017).

## Tools and Technologies to Enhance Integration of Thrips Resistance in Peanut

Availability of novel tools and resources could facilitate a renewed interest in breeding for thrips resistant peanut cultivars, a few of them are discussed below.

### Thrips Screening

Improvement in traditional and novel breeding approaches will result in hundreds and hundreds of peanut genotypes that need to be screened for thrips resistance. Until recently, most phenotypic evaluations for thrips resistance have focused on field screening for foliar feeding damage. Recently, a few studies have performed laboratory experiments to examine resistance to thrips in peanut (Shrestha et al., 2013; Sundaraj et al., 2014). These assays were used to evaluate both behavior such as preference and end-point parameters such as thrips feeding damage and biological fitness. These tests need to be conducted for weeks if not months, and could be laborious especially if there are many genotypes involved. Until recently, there was no automated high-throughput screening tool available for thrips, but have been available for hemipteran insects such as aphids. Recently, one such automated thrips-resistance screening tool has become available (Thoen et al., 2016). Thoen et al. (2016) used an automated video monitoring parallel choice test platform to screen ∼350 *Arabidopsis* accessions within a week. By using this assay, they could measure parameters that are obtained in traditional choice tests such as leaf damage and number of nymphs produced within a certain time. In addition to these usual parameters, they were also able to get information pertaining to thrips behavior on the host plant using a behavior analyzing software Ethovision^®^ XT 10. The parameters estimated included time spent on the foliage, time not moving on the foliage, time not moving, distance moved, and movement velocity. Assessing these parameters for peanut genotype screening against thrips will provide more insights into thrips-peanut plant interactions facilitate faster and better screening for resistance. Electronic nose is another tool that could be used for quick and efficient screening for thrips resistance in peanut. This technique was found to be useful to discriminate Western flower thrips resistant genotypes from susceptible chrysanthemum genotypes effectively using headspace volatile profiles following simulated thrips feeding and thrips feeding bioassays (McKellar et al., 2005). The usefulness of such a technique needs to be explored in screening for thrips resistance in peanut.

### Marker Assisted Selection and Single Nucleotide Polymorphisms

Genomic tools have been used for crop breeding to develop improved varieties. In particular, the application of trait-linked DNA markers to facilitate trait selection (Marker Assisted Selection – MAS) for crop improvement have proved successful for major crops. MAS uses trait-linked markers, instead of trait itself, has the advantage of eliminating plants with undesirable gene combinations, allowing the breeder to concentrate on a lower number of better lines. It has proven successful for cultivar improvement on many major crops. Peanut, however, has lagged behind the major crops due to the intrinsic narrow genetic variability delaying the identification of markers useful for selection. With a concerted effort by the Peanut Genome Initiative, an international group of scientists, genetic and genomic tools became available to the community and made marker assisted selection a reality in for peanut breeding (Stalker and Mozingo, 2001; Wang et al., 2018). Various marker systems have been developed throughout the years, following technology development (for a comprehensive review, see Wang et al., 2018). Recently, the most significant achievement was the sequence of the genome of the two wild progenitors of peanut, *A. duranensis* and *A. ipaensis* (Bertioli et al., 2016) and the development of a publicly available genotyping tool: a chip with nearly 60,000 SNPs (Clevenger et al., 2017; Pandey et al., 2017).

To date, only one study was conducted that showed correlation of a molecular marker with a peanut virus vector, *Aphis craccivora*, which transmits *Groundnut rosette virus* (Genus *Umbravirus*; Family *Tombusviridae*) (Herselman et al., 2004). In other crops (but not peanuts), markers have been associated with resistance to thrips (e.g., cowpea Lucas et al., 2012; pepper Maharijaya et al., 2015; tomato, Escobar-Bravo et al., 2017). For peanut, we still need to understand better the nature of thrips resistance and develop molecular markers.

Research on *Orthotospovirus* resistance, on the other hand, have seen more advances. For instance, TSWV host resistance has been established as a major factor to reduce disease risk, and therefore breeding for resistance has become a major goal in the breeding programs in the United States. Phenotypic selection for TSWV is inaccurate as field resistance expression varies significantly from year to year, depending on the environment (Culbreath et al., 2010; Tseng et al., 2016). *In vitro* transmission is also not considered reliable as it bypasses some possible plant defense responses (Zhao et al., 2018). Therefore, breeding for resistance to orthotospoviruses such as TSWV could greatly be facilitated by implementation of MAS in breeding programs. The first population, major QTLs were found in LG A01, and in the second study, 11 minor QTLs were found in different regions of the genome (Khera et al., 2016; Pandey et al., 2017). Work with another population, Florida EPTM^13^ x Georgia Valencia revealed major QTLs on LG01, and markers tightly linked to TSWV resistance (Tseng et al., 2016; Zhao et al., 2018). Association genetics using these markers on the United States minicore collection, however, did not show association between TSWV resistance and the markers (Li et al., 2018; Zhao et al., 2018). This is probably because the resistance allelic region to which the marker is associated is from a unique source, which is not present in the United States peanut mini core gene pool. This well documented study flags caution on the use of correct and validated molecular markers on genotypes with the same allele variants.

All the studies described above were conducted mainly using Simple Sequence Repeat (SSR) markers, which are costly, time consuming and not very abundant. New genotyping methods using Single Nucleotide Polymorphism (SNPs) are currently available (Bertioli et al., 2014; Clevenger et al., 2017; Pandey et al., 2017) and, together with more precise genotyping, are likely to speed the discovery of tightly linked markers and consequently, the implementation of MAS for in peanut breeding against thrips and viruses. MAS is not the only, or the main one, but it is undoubtedly, a very useful tool in the breeder’s tool box.

### Transgenic Thrips Resistance and RNAi

Peanut transformation was first reported by Brar et al. (1994) but no resistance tests were performed. Peanut has been transformed with *cry* genes derived from *Bacillus thuringiensis* (Berliner) that confer resistance to several insects, especially of the order Lepidoptera (Krishna et al., 2015). However, to our knowledge, no transgenic peanut has been tested or found to be resistant to thrips. In other crops, transgenesis has been successful to transfer resistance to thrips: resistance to western flower thrips has been engineered in potato plants (Outchkourov et al., 2004). Members of inhibitors of cysteine and aspartic proteases *viz*., stefin, potato cystatin, equistatin, and cystatin were fused and made into a functional unit and expressed in potato plants. The plants containing these multidomain proteins had fewer larvae and adults when compared with non-transgenic control plants (Outchkourov et al., 2004).

Resistance to orthotospoviruses in transgenic peanut has been achieved multiple times. In the United States and in India, both Valencia and runner genotypes were transformed with TSWV/GBNV N-gene or coat protein-based constructs, and pathogen-derived resistance was achieved in these transformants. Transformed peanut seedlings provided substantial field or *in vitro* resistance against TSWV and GBNV (Li et al., 1997; Magbanua et al., 2000; Yang et al., 2004; Rao et al., 2013). Mehta et al. (2013) also obtained transgenic peanut resistant to *Peanut stem necrosis virus*. So, the potential for viral resistance is very significant.

RNA interference has been deployed to provide resistance against a wide range of organisms including viruses and insects (Whyard et al., 2009; Gan et al., 2010; Zhang et al., 2013). In this process, the dsRNA pertaining to an invading organism is degraded into short interfering (si) RNAs (20–23 nucleotides long) with the help of an enzyme complex, and subsequently the siRNAs block the mRNA translation (Fire et al., 1998; Fire, 2007). RNAi and its usefulness have not been demonstrated in peanuts against viruses and/or thrips. Nevertheless, the usefulness of RNAi has been demonstrated with *F. occidentalis*, wherein double stranded RNA (dsRNA) pertaining to an important enzyme (vacuolar ATP-Synthase or V-ATPase) was silenced, following which a significant reproductive fitness was observed in *F. occidentalis* (Badillo-Vargas et al., 2015). Advancements in thrips genomics and transcriptomics has led to identification of various developmental genes associated with western flower thrips and tobacco thrips in recent years (Schneweis et al., 2017; Shrestha et al., 2017). The usefulness of these genes should first be validated through *in vitro* assays, and further their *in planta* expression and effectiveness against thrips should be attempted to examine the usefulness of RNAi as a management option. Though consumer preference will keep transgenic peanut from coming to the marketplace anytime soon, available transgenic technology does offer some very interesting research possibilities. Technologies such as RNAi could be modified in certain ways to circumvent plant-based transgene delivery. In fact, the versatility of RNAi has been demonstrated in other cropping systems by mere exogenous application. For instance, Gogoi et al. (2017) observed that exogenous application of *Zucchini yellow mosaic* (Genus *Potyvirus*; Family *Potyviridae*) virus specific dsRNA molecules when applied to tomato plants were detected in the plants up to 14 days post application, and they were also detected in phloem feeding insects such as aphids and whiteflies up to 10 days post application (Gogoi et al., 2017). In another study, exogenous application of dsRNA specific to *Tobacco mosaic virus* (TMV) (Genus *Tobamovirus*; Family *Virgaviridae*) limited the systemic movement of TMV and conferred resistance to TMV inoculation (Konakalla et al., 2016). RNAi is very specific to the target organism and should greatly minimize non-target effects, and therefore could be highly useful and acceptable especially when an exogenous application strategy is considered. This RNAi strategy (exogenous application) will help alleviate the regulatory hurdles and consumer concerns associated with peanut or any other crop. However, this technology is just emerging, its suitability and its commercial viability for peanut production need to be developed and explored in greater depth. Nevertheless, it is promising and reassuring that thrips management in peanut may not have to exclusively rely on spraying insecticides in the long run. Stacking thrips resistance with pathogen resistance could have multifaceted benefits to peanut growers worldwide.

## Conclusion

Even though thrips-transmitted viruses have garnered substantial attention in the last two decades, it is abundantly clear that thrips continue to cause damage by direct feeding in peanut production systems throughout the world. Host-resistance to orthotospoviruses is the main management option adopted in peanut, but it is used in conjunction with cultural practices and insecticide applications targeted at thrips. Unlike other crops such as tomato and pepper where the resistance is governed by a major gene, neither is resistance to orthotospoviruses complete nor is the mechanism of resistance known in peanut (Shrestha et al., 2013; Sundaraj et al., 2014). Studies suggest that virus resistance in peanut may not be an exclusive result of host–virus interactions, but could also be influenced by peanut–thrips interactions (do Nascimento et al., 2006; Sundaraj et al., 2014; Shrestha et al., 2015). *A. hypogaea*, an allotetraploid, has a very narrow genetic base and has little to no inherent resistance to thrips and/or viruses transmitted by them (Ratnaparkhe et al., 2011). Numerous studies have documented that wild species source of resistance for thrips and orthotospoviruses. Therefore, it may be possible to incorporate resistance to both thrips and the virus simultaneously. Differences in ploidy level and the subsequent dilution effect in resistance introgression due to backcrossing represent the biggest hurdle for using wild species in breeding for resistance. This obstacle could be overcome by using novel strategies such as development of synthetic tetraploids (Leal-Bertioli et al., 2015). Molecular markers could also be potentially used to link thrips and virus resistance (Bertioli et al., 2014). Stacking resistance against thrips and orthotospoviruses could reduce selection pressure on the virus itself, delay or prevent evolution of highly virulent strains, prolong the usefulness of resistant cultivars, reduce insecticide usage and allied non-target effects, and promote sustainability in peanut production.

## Author Contributions

RS developed the idea and contributed to writing. MA co-developed the idea and contributed to writing. P-CL conducted the experiments included in manuscript and contributed to writing. AC contributed to writing. ST was a collaborator on *Arachis diogoi* work and contributed to writing. SL-B contributed to writing.

## Conflict of Interest Statement

The authors declare that the research was conducted in the absence of any commercial or financial relationships that could be construed as a potential conflict of interest.

## Footnotes

1 https://www.epa.gov/sites/production/files/2015-04/documents/neonicotinoid-new-use.pdf
